# Supplementary material for: Bacteriophage targeting microbiota alleviates non-alcoholic fatty liver disease induced by high alcohol-producing Klebsiella pneumoniae
Source: Nat Commun. 2023 Jun 3;14:3215. doi: 10.1038/s41467-023-39028-w (PMC10239455; doi:10.1038/s41467-023-39028-w)
Supplement: Supplementary file 1 — Supplementary Information [file 41467_2023_39028_MOESM1_ESM.pdf]

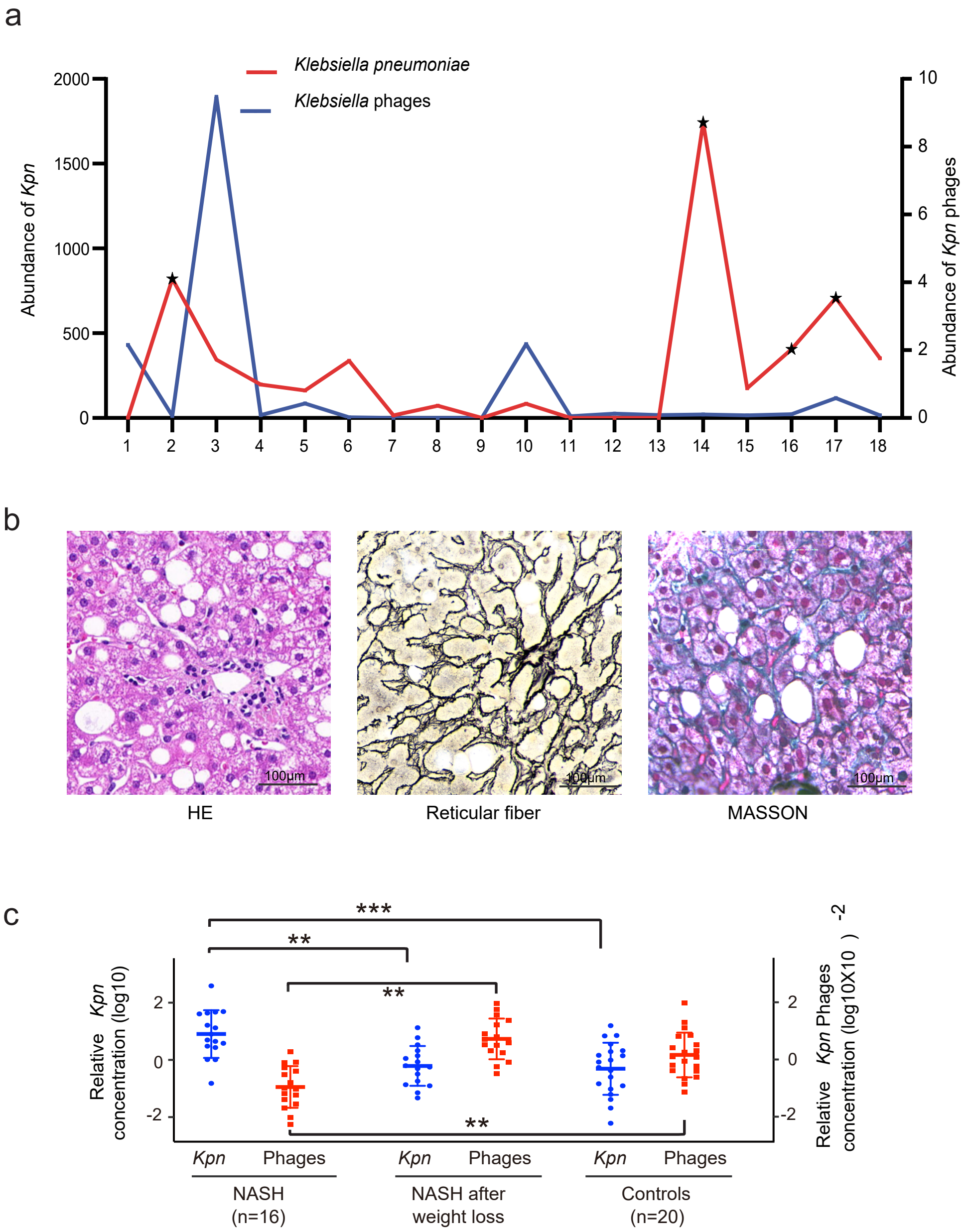

Figure S1. Abundances of *Klebsiella pneumoniae* and *Klebsiella* phages in fecal samples. (a) Abundances of *K. pneumoniae* and *Klebsiella* phages in fecal samples collected from a patient with non-alcoholic steatohepatitis (NASH) and recurrent pancreatitis. The black asterisks denote pancreatitis attack periods of the patient. (b) Percutaneous liver biopsy of the patient. Histological stainings (HE, Reticular fiber and Masson) showed that there were increases in fatty droplets, infiltration of inflammatory cells and deposition of collagens in sections of the liver tissues, suggesting that the NASH patient was in liver fibrosis stage. (c) Relative concentrations of *K. pneumoniae* and *Klebsiella* phages in fecal samples collected from NASH patients, NASH patients after weight loss and controls. Values are expressed as the mean ± SD. One-way ANOVA, *P*-value < 0.05 (*), 0.01 (**) or 0.001 (***).
