# Supplementary material for: Bacteriophage targeting microbiota alleviates non-alcoholic fatty liver disease induced by high alcohol-producing Klebsiella pneumoniae
Source: Nat Commun. 2023 Jun 3;14:3215. doi: 10.1038/s41467-023-39028-w (PMC10239455; doi:10.1038/s41467-023-39028-w)
Supplement: Supplementary file 3 — Description of Additional Supplementary Files [file 41467_2023_39028_MOESM3_ESM.pdf]

Description of Additional Supplementary Files

File name: Supplementary Data 1

Descriptions: Characteristics of clinical NASH patients and *K. pneumoniae* isolates

File name: Supplementary Data 2

Descriptions: The bacteria host range of phage phiW14

File name: Supplementary Data 3

Descriptions: The putative open reading frames of phage phiW14

File name: Supplementary Data 4

Descriptions: The identified proteins of phage phiW14

File name: Supplementary Data 5

Descriptions: Differential expression genes of liver tissues in endo-AFLD mice

File name: Supplementary Data 6

Descriptions: Differential metabolites of feces in endo-AFLD mice

File name: Supplementary Data 7

Descriptions: Primers used in this study
