# Supplementary material for: Bacteriophage targeting microbiota alleviates non-alcoholic fatty liver disease induced by high alcohol-producing Klebsiella pneumoniae
Source: Nat Commun. 2023 Jun 3;14:3215. doi: 10.1038/s41467-023-39028-w (PMC10239455; doi:10.1038/s41467-023-39028-w)
Supplement: Supplementary file 5 — Reporting Summary [file 41467_2023_39028_MOESM5_ESM.pdf]

nature portfolio

Corresponding author(s): Jing Yuan

Last updated by author(s): May 23, 2023

Reporting Summary

Nature Portfolio wishes to improve the reproducibility of the work that we publish. This form provides structure for consistency and transparency in reporting. For further information on Nature Portfolio policies, see our Editorial Policies and the Editorial Policy Checklist.

Statistics

For all statistical analyses, confirm that the following items are present in the figure legend, table legend, main text, or Methods section.

n/a	Confirmed	
☐	☒	The exact sample size (*n*) for each experimental group/condition, given as a discrete number and unit of measurement
☐	☒	A statement on whether measurements were taken from distinct samples or whether the same sample was measured repeatedly
☐	☒	The statistical test(s) used AND whether they are one- or two-sided<br>*Only common tests should be described solely by name; describe more complex techniques in the Methods section.*
☐	☒	A description of all covariates tested
☐	☒	A description of any assumptions or corrections, such as tests of normality and adjustment for multiple comparisons
☐	☒	A full description of the statistical parameters including central tendency (e.g. means) or other basic estimates (e.g. regression coefficient) AND variation (e.g. standard deviation) or associated estimates of uncertainty (e.g. confidence intervals)
☐	☒	For null hypothesis testing, the test statistic (e.g. *F*, *t*, *r*) with confidence intervals, effect sizes, degrees of freedom and *P* value noted<br>*Give P values as exact values whenever suitable.*
☒	☐	For Bayesian analysis, information on the choice of priors and Markov chain Monte Carlo settings
☒	☐	For hierarchical and complex designs, identification of the appropriate level for tests and full reporting of outcomes
☒	☐	Estimates of effect sizes (e.g. Cohen's *d*, Pearson's *r*), indicating how they were calculated

Our web collection on statistics for biologists contains articles on many of the points above.

Software and code

Policy information about availability of computer code

Data collection	No software was used .
Data analysis	MEGA (version 11.0.13), hisat2 (version 2.1.0), QIIME2 (version 2018.2), Trimmomatic (version 0.39), DESeq (version 1.34.1), DESeq2 (version 1.22.2), R package (version 3.6.2), SPSS (version 20.0)

For manuscripts utilizing custom algorithms or software that are central to the research but not yet described in published literature, software must be made available to editors and reviewers. We strongly encourage code deposition in a community repository (e.g. GitHub). See the Nature Portfolio guidelines for submitting code & software for further information.

Data

Policy information about availability of data

All manuscripts must include a data availability statement. This statement should provide the following information, where applicable:

- Accession codes, unique identifiers, or web links for publicly available datasets
- A description of any restrictions on data availability
- For clinical datasets or third party data, please ensure that the statement adheres to our policy

The whole genome sequence of K. pneumoniae phage phiW14 was deposited in the NCBI (https://www.ncbi.nlm.nih.gov/) under accession number OK655936. The raw sequence data of transcriptome were deposited in the Genome Sequence Archive (GSA, https://ngdc.cncb.ac.cn/gsub/) under PRJCA008648/CRA006357. The raw sequence data of metabolome were deposited in the Open Archive for Miscellaneous Data (OMIX, https://ngdc.cncb.ac.cn/omix/) under PRJCA008973/OMIX001072. Source data are provided with this paper.

Research involving human participants, their data, or biological material

Policy information about studies with human participants or human data. See also policy information about sex, gender (identity/presentation), and sexual orientation and race, ethnicity and racism.

Reporting on sex and gender	In this study, all subjects' sex were consistent with their gender.The patient diagnosed with NASH accompanied by recurrent pancreatitis was a 55-year-old man; in groups of 16 patients with NASH, 11 subjects were male, and 5 subjects were female; in groups of 20 healthy controls, 9 subjects were male, and 11 subjects were female.As the HiAlc Kpn was the major object, we did not performed the sex- and gender-based analysis.
Reporting on race, ethnicity, or other socially relevant groupings	All participants are Chinese varied from 30 to 68 years old. And all 16 patients were diagnosed as NASH.
Population characteristics	See above.
Recruitment	All participants were recruited voluntarily and randomly.
Ethics oversight	All human participants signed the informed consent form in this study. This study was approved by the Medical Ethics Committee of the Capital Institute of Pediatrics and carried out by the licensed individual with license number SHERLL2021032.

Note that full information on the approval of the study protocol must also be provided in the manuscript.

Field-specific reporting

Please select the one below that is the best fit for your research. If you are not sure, read the appropriate sections before making your selection.

☒ Life sciences ☐ Behavioural & social sciences ☐ Ecological, evolutionary & environmental sciences

For a reference copy of the document with all sections, see nature.com/documents/nr-reporting-summary-flat.pdf

Life sciences study design

All studies must disclose on these points even when the disclosure is negative.

Sample size	In animal assays, all mice were randomly divided into groups, 6 mice in each group. This sample size (n=6) is avalible for statistical analysis.
Data exclusions	No data was excluded in this study.
Replication	All murine models were conducted with 3 independent experiments (n=6 mice/group).
Randomization	All mice/ samples were randomly divided into groups
Blinding	Investigators were blinded to group allocation during data collection and analysis.

Reporting for specific materials, systems and methods

We require information from authors about some types of materials, experimental systems and methods used in many studies. Here, indicate whether each material, system or method listed is relevant to your study. If you are not sure if a list item applies to your research, read the appropriate section before selecting a response.

Materials & experimental systems

n/a	Involved in the study
x	Antibodies
x	Eukaryotic cell lines
x	Palaeontology and archaeology
	x Animals and other organisms
x	Clinical data
x	Dual use research of concern
x	Plants

Methods

n/a	Involved in the study
x	ChIP-seq
x	Flow cytometry
x	MRI-based neuroimaging

Animals and other research organisms

Policy information about studies involving animals; ARRIVE guidelines recommended for reporting animal research, and Sex and Gender in Research

Laboratory animals	SPF male C57BL/6J mice (6-7 weeks); Germ-free male C57BL/6J mice (6-7 weeks).
Wild animals	No wild animals were used in this study.
Reporting on sex	All mice used in this study were male; As the HiAlc Kpn was the ma J or object, we did not performed the sex- and gender-based analysis
Field-collected samples	No sample was collected from the field
Ethics oversight	All mouse experiments were approved by the Medical Ethics Committee of the Capital Institute of Pediatrics and carried out by the licensed individual with license number DWLL2021009

Note that full information on the approval of the study protocol must also be provided in the manuscript.
